# N-doped MXenes for tribological applications: A high-throughput DFT dataset

**DOI:** 10.1038/s41597-026-07236-w

**Published:** 2026-04-17

**Authors:** Mingyang Xu, Yu Gao, Wenhao He, Zhongrong Geng, Qian Meng, Zhibin Lu

**Affiliations:** 1 School of Materials Science and Engineering, Lanzhou Jiaotong University, Lanzhou, 730070 China; 2 State Key Laboratory of Solid Lubric ation, Lanzhou Institute of Chemical Physics, Chinese Academy of Sciences, Lanzhou, 730000 China; 3 Scientific Data Center, Lanzhou Institute of Chemical Physics, Chinese Academy of Sciences, Lanzhou, 730000 China

## Abstract

MXenes have emerged as a prominent class of two-dimensional materials in the field of solid lubrication, and nitrogen doping has recently been identified as an effective strategy for tailoring their properties. Herein, we present a comprehensive dataset of 210 pristine and N-doped MXene structures with detailed electronic, thermodynamic, and tribological properties obtained through density functional theory (DFT). Selected properties are visualized to illustrate doping effects. This dataset not only provides fundamental insights into the interfacial behavior of MXenes but also establishes a foundational benchmark for the machine-learning-driven design of reliable, low-friction N-doped MXene coatings.

## Background & Summary

Among various two-dimensional (2D) materials, MXenes have attracted significant interest due to their tunable interlayer spacing and weak interlayer interactions, making them promising for lubrication applications. Initially reported in 2011 by Gogotsi and Barsoum^1^, MXenes represent a family of 2D transition metal carbides, nitrides, or carbonitrides. They are derived from their parent MAX phases (where A is typically a group 13 or 14 element) by selective etching of the A layers. MXenes have the general formula M_n+1_X_n_T_x_ (n = 1, 2, 3, 4), where M denotes early transition metals (e.g., Ti, V, Nb, Ta, Mo), X is C or N, and T represents surface terminations such as -O, -OH or -F that arise from the synthesis process^2^. Owing to inherent chemical versatility of the M and X sites and the tunability of surface terminations, the MXene family exhibits unique compositional and structural diversity, making them promising for a wide range of applications, including solid lubrication.

Introducing heteroatoms, particularly nitrogen, has emerged as a powerful strategy to tune the properties of 2D materials. For instance, nitrogen doping enhances the chemical activity of graphene, facilitating its adsorption on sliding surfaces and improving the stability of tribofilms^3^. Similarly, N-doped diamond-like carbon (DLC) films exhibit more stable tribological behavior compared to their undoped counterparts^4^. Beyond tribology, N-doping improves the electrical conductivity of MoS_2_ for electrochemical applications^5^. Recently, this strategy has been extended to MXenes. Chen *et al*.^6^ demonstrated that zwitterionic polymer-functionalized N-doped MXene nanosheets act as effective aqueous lubricant additives, substantially reducing friction and wear. Le *et al*.^7^ synthesised N-doped Ti_3_C_2_T_x_ and observed enhanced adsorption performance and electrical conductivity upon doping. While these experimental studies underscore the macroscopic benefits of nitrogen incorporation, the underlying atomistic mechanisms, including the effects of doping on interlayer interactions and frictional response, remain largely unexplored.

Density functional theory (DFT) simulations are ideally suited to bridge this knowledge gap by providing atomic-level insights. DFT has been widely employed to compute the tribological properties of materials, often by exploring the potential energy surface (PES) along sliding paths. Cao *et al*.^8^ utilized the charge density evolution to characterize static negative friction coefficient, while Jeong *et al*.^9^ identified nearly frictionless paths in black phosphorus/graphene and black phosphorus/graphene oxide systems, suggesting the possibility of superlubricity. However, most previous studies have focused on the optimal (lowest-energy) sliding paths, whereas the worst-case sliding path, which is critical for assessing material reliability under harsh conditions has received little attention. Recently, Feng *et al*.^10^ addressed this gap by investigating tribological properties along the worst-case sliding path for homojunctions of nine 2D materials, identifying promising solid lubricants and providing theoretical guidance for experiments. While the aforementioned DFT studies have provided valuable insights into friction mechanisms in 2D materials, their application to nitrogen-doped MXenes is severely constrained by the high computational cost required to obtain reliable, high-quality data, which has precluded systematic investigations.

To overcome this limitation, we have constructed a comprehensive dataset comprising 210 structures, including 126 N-doped and 84 pristine configurations derived from five MXenes materials (Nb_2_C, V_2_C, Sc_2_C, Ta_2_C, and Mo_2_C), with four doping sites. The dataset systematically integrates key properties calculated by DFT, including sliding energy barrier (*ΔE*) and friction force (*f*) along the worst-case sliding path on the PES as a function of interlayer spacing, as well as key electronic (total density of states TDOS, band gap *E*_*g*_), mechanical (Young’s modulus *E*, shear modulus *G*, Poisson’s ratio *υ*), and thermodynamic parameters (binding energy BE, formation energy *E*_*f*_). By consolidating high-throughput computational data on both pristine and N-doped MXenes, this resource provides critical benchmarks for evaluating their mechanical stability and operational limits under harsh sliding conditions. Furthermore, it establishes a foundational dataset for machine learning models aimed at screening and designing robust, N-doped MXene coatings with predictable and reliable tribological properties.

## Methods

### Structural construction

**Fig. 1:**
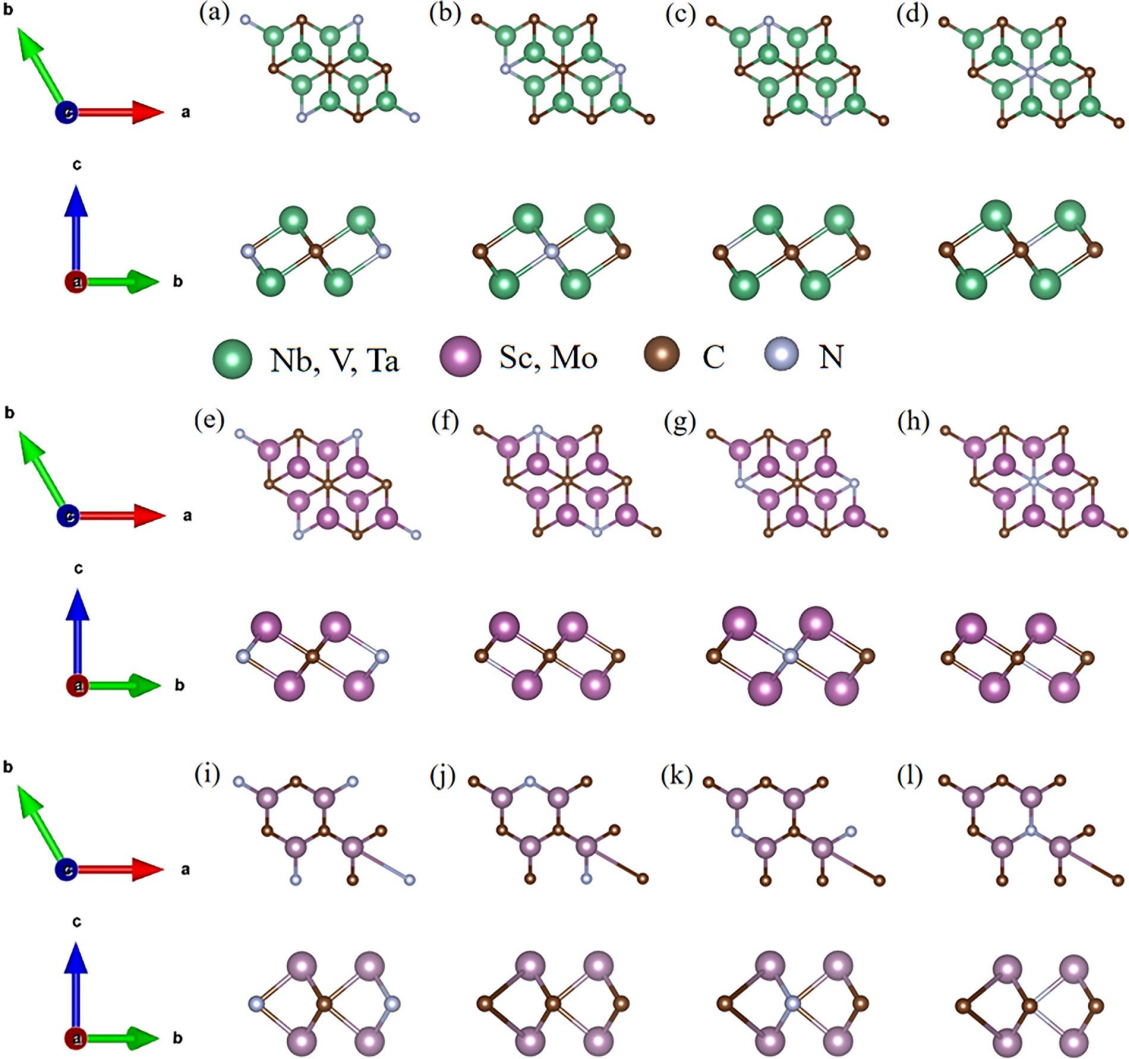
Representative atomic structures of the four distinct doping sites in two classes of MXenes. (**a**–**d**) Sites 1–4 in Nb_2_C (Nb = V, Ta) (**e**–**h**) Sites 1–4 in Sc_2_C (**i**–**l**) Sites 1–4 in Mo_2_C.

The initial bulk structures for Nb_2_C, V_2_C, Sc_2_C, Ta_2_C, and Mo_2_C were obtained from the the open-access Materials Project database (https://legacy.materialsproject.org)^11^. Each structure was retrieved by searching the database using the material’s chemical formula (e.g., “Nb2C”) and selecting the corresponding entry. The specific Materials Project IDs for all structures are included in the deposited dataset for full traceability. We deliberately omitted surface terminations to focus on the intrinsic effects of N-doping on the atomic and electronic structure of the MXene host, thereby excluding the interference of surface functional groups. Considering structural stability, Nb_2_C, V_2_C, Sc_2_C, and Ta_2_C were modeled with octahedral coordination, while Mo_2_C was modeled with trigonal-prismatic coordination^12^. However, we acknowledged that, compared to experimentally synthesized MXenes, which typically possess various surface functional groups, our termination-free models indeed have certain limitations. Nevertheless, this dataset can serve as a foundational baseline for future studies incorporating explicit surface terminations. A 2 × 2 × 1 supercell was constructed for each material, followed by substitutional doping. To simulate the frictional process, the bulk structures downloaded from the Materials Project database were cleaved along the [001] crystallographic plane to obtain single-layer MXene slabs. Subsequently, the monolayers were stacked in an AA configuration to construct bilayer systems. Finally, a vacuum layer of 15 Å was added in the direction perpendicular to the sliding direction to eliminate unphysical interactions between periodic images. The atomic structures of the doped materials are depicted in Fig. 1.

### Computational details

All DFT calculations were performed with the Vienna Ab initio Simulation Package (VASP)^13^. The exchange-correlation energy was estimated with the Perdew-Burke-Ernzerhof (PBE) functional within the generalized gradient approximation (GGA)^14^, with DFT-D3 method, employed to describe the van der Waals (vdW) interactions, which are crucial for accurately modeling interlayer binding and sliding^12,15–17^. A plane-wave basis set with a cutoff energy of 400 eV was used. The Brillouin zone was sampled with k-point meshes of 5 × 5 × 6 for monolayer relaxation and 5 × 5 × 1 for bilayer systems, with the k-point convergence tests detailed in Fig. S1 of the Supporting Information. Tribological properties were evaluated using the self-developed First-Principles High-Throughput Computing Platform for Solid Interface Friction Properties (LICP-FPHTC-Platform)^18^.

The relaxed bilayer structures served as the initial configurations for tribological calculations. To map the PES, the upper layer was displaced in a grid of 6 steps along both the *x* and *y* directions at different interlayer distances, with varying numbers of steps (6 or 12) along the *z* to sample different interlayer distances. The convergence of this in-plane grid was verified, as detailed in the Supporting Information (Fig. S2). A series of sampling points along the *z* were determined by moving upward and downward by specific distances from the equilibrium interlayer distance, which was obtained from the fully relaxed bilayer system. The energies of the system at these different interlayer distances were then used to compute *ΔE* along the worst-case sliding path, which is defined as the path traversing the maximum and minimum energy points on the PES during interfacial sliding. The *ΔE* and *f*^19^ according to Eqs. (1) and (2), both normalized based on the 2 × 2 × 1 supercell bilayer system:

1 $$\Delta {E}={{E}}_{{\max }}-{{E}}_{{\min }}$$

2 $${f}=\frac{\Delta {E}}{{d}}$$

where *d* is the slip distance, *E*_*max*_ and *E*_*min*_ are the maximum and minimum energy points along the slip path, respectively. The *f* was initially obtained in units of eV/Å from Eq. (2), these values were converted to nanonewtons (nN) using the conversion relationship 1 eV/Å = 1.602 nN.

To further elucidate the site-specific doping effects observed in the electronic structure, we performed differential charge density^20^ and Bader charge analyses^21^. We also performed spin-polarized test calculations for all five MXene systems (one representative doping site each). The total energy differences between spin-polarized and non-spin-polarized calculations were negligible (see Table S1 in Supporting Information), confirming that spin polarization does not significantly affect the total energies or derived properties in this study. Therefore, spin polarization and spin-orbit coupling were not included in the production calculations. Although test calculations confirm their effects are negligible for the properties studied, we acknowledge this as a limitation of the current work.

The elastic stiffness matrix was calculated using DFT on the bulk structures with convergence settings of EDIFF = 1 × 10^−6^ eV and EDIFFG = −0.01 eV/Å. The resulting elastic stiffness matrix was then input into the ELATE application (https://progs.coudert.name/elate)^22^ to derive the mechanical properties (*E*, *G*, *υ*), adopting the Hill scheme from the Averaging scheme options provided. The BE^19^ and *E*_*f*_^23^ were calculated as follows, both normalized based on the 2 × 2 × 1 supercell bilayer system:

3 $$BE={E}_{b}-2\times {E}_{s,doped}$$

4 $${E}_{f}={E}_{s,doped}-{E}_{s,undoped}-{E}_{N}+{E}_{C}$$

where *E*_*b*_ is the total energy of the bilayer structure, *E*_*s, doped*_ and *E*_*s, undoped*_ are the total energies of the doped and pristine monolayers, respectively. *E*_*N*_ and *E*_*C*_ represent the chemical potential of nitrogen and carbon atoms. TDOS and *E*_*g*_ were obtained using the vaspkit code^24^.

## Data Records

The complete dataset, comprising all atomic structures and corresponding properties for both pristine and N-doped MXenes, is available in CSV format from the Science Data Bank^25^. The data records include fundamental structural information (crystal system, space group, lattice parameters, total surface area, and unit cell volume), interface-specific properties (interlayer contact area, interlayer distance, and total system height) and all DFT-calculated properties (*f*, *ΔE*, *E*_*b*_, *E*_*s*_, *E*, *G*, *υ*, BE, *E*_*f*_, and *E*_*g*_). A detailed description of each column header is provided in Table 1.

**Table 1:** Key parameters contained within the CSV file.

Parameters	Description
Materials Project IDs	Materials Project IDs of the structures
S_total_ (Å^2^)	total surface area of the unit cell
S_int_ (Å^2^)	interlayer contact area in bilayer system
V (Å^3^)	total volume of the unit cell
d_total_ (Å)	total height of the system
d_layer_ (Å)	interlayer spacing
a (Å)	lattice constants a
a/c	lattice constants a/c
E_b_ (eV)	total energy of bilayer system
E_s_ (eV)	total energy of monolayer system
E_f_ (eV)	formation energy
BE (eV)	binding energy
E_g_ (eV)	band gap
E (GPa)	Young’s modulus
G (GPa)	Shear modulus
υ	Poisson ratio
E_N_ (eV)	chemical potential of the N atom
E_C_ (eV)	chemical potential of the C atom
ΔE (eV)	slip energy barrier
f (nN)	friction force

## Data Overview

**Fig. 2:**
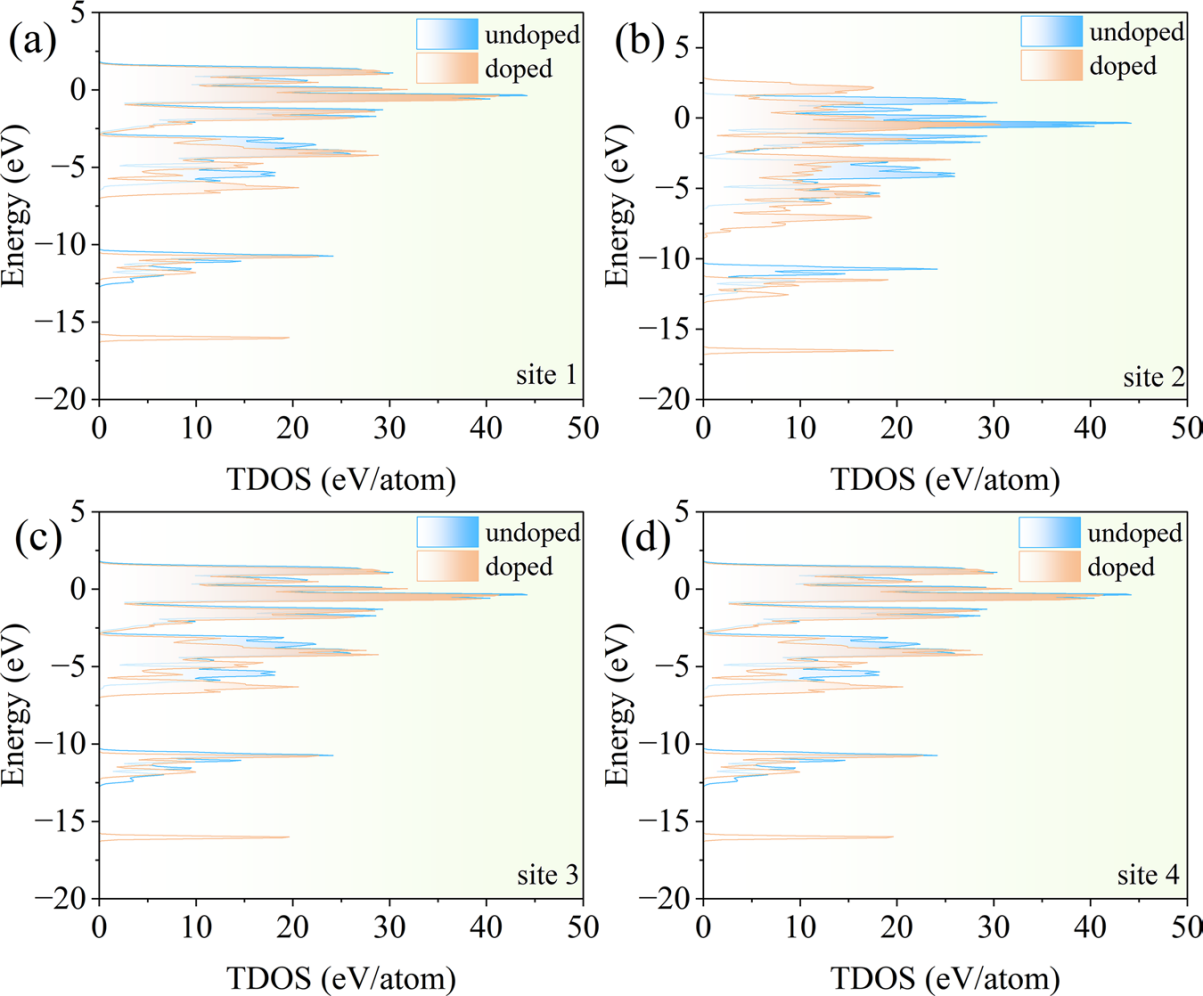
Comparison of TDOS for V_2_C before and after doping at different sites. (**a**) site 1 (**b**) site 2 (**c**) site 3 (**d**) site 4. The blue and orange curves represent the TDOS of the pristine and N-doped structures, respectively.

**Fig. 3:**
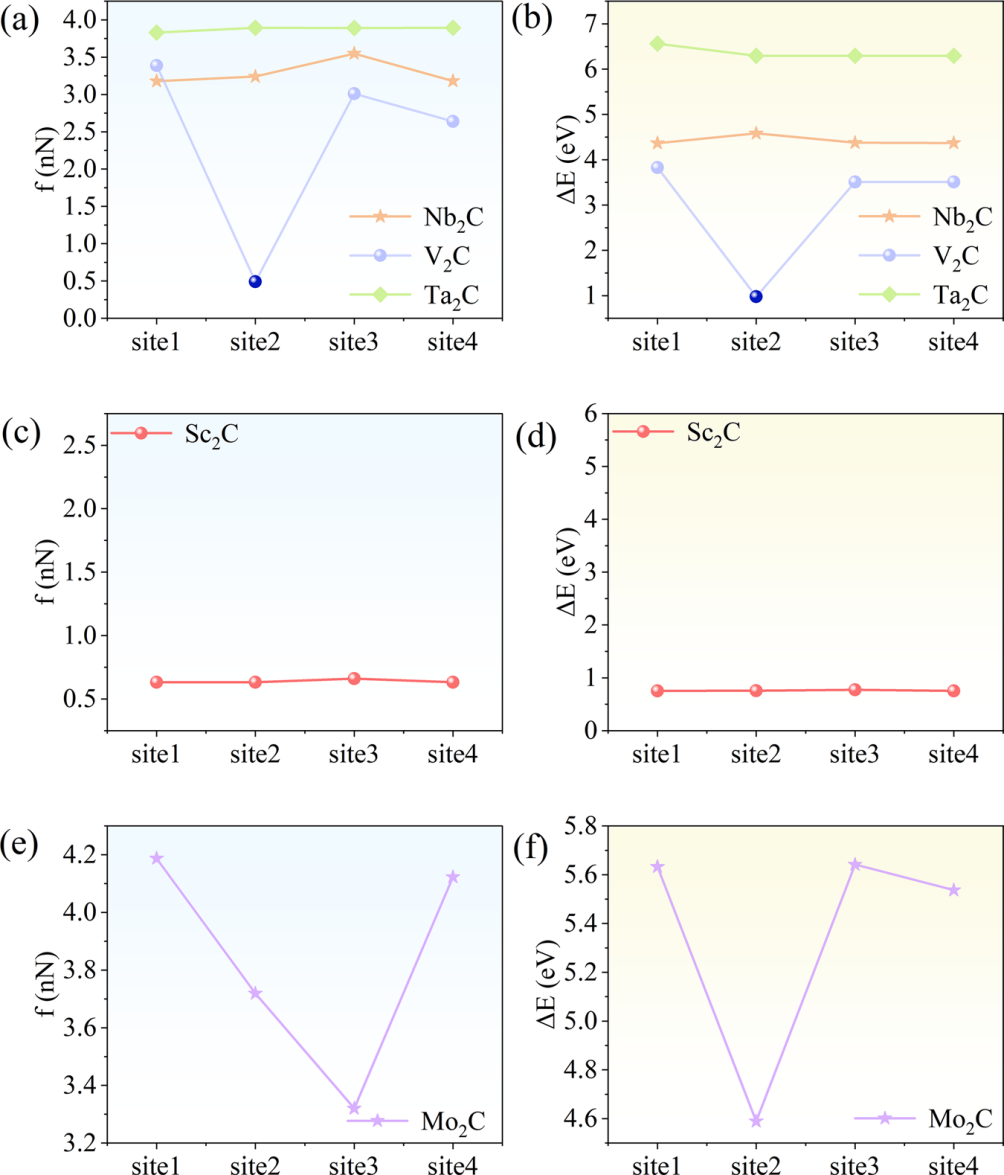
Tribological properties of N-doped MXenes across various doping sites. (**a**) *f* and (**b**) *ΔE* for Nb_2_C (orange), V_2_C (blue), and Ta_2_C (green). (**c**) *f* and (**d**) *ΔE* for Sc_2_C. (**e**) *f* and (**f**) *ΔE* for Mo_2_C.

The dataset integrates tribological, electronic, mechanical, and thermodynamic properties derived from DFT calculations. For Mo_2_C, a notable minimum in *f* is observed at doping site 3, while *ΔE* exhibits pronounced variations at site 2 (Fig. 3e,f). This discrepancy arises from changes in the sliding path, which alters the effective slip distance. Electronic structure analysis of V_2_C reveals clear site-specific doping effects: doping at site 2 leads to a reduced and more uniform TDOS distribution near the Fermi level, whereas other sites show increased and discontinuous features (Fig. 2). Such site-specific effects are not unique to MXenes but represent a general phenomenon in doped 2D materials, as demonstrated by Hazarika and Kalita^26^ in MgO monolayers. To understand the origin of this site dependence, we performed differential charge density and Bader charge analyses (see Supplementary Information, Fig. S3 and Table S2).

## Technical Validation

To validate the reliability of our computational methodology, we compared our calculated mechanical properties with previously reported DFT results. We note that direct comparison requires ensuring the same crystal structure; therefore, we selected literature data for Nb_2_C^27^, that corresponds to the same space group as our models. As shown in Table 2, our results are in excellent agreement with Ref. ^27^, with deviations below 8%. The minor remaining discrepancies can be attributed to differences in computational software and pseudopotentials. This consistency confirms the reliability of our computational setup. In the case of Mo_2_C, our results demonstrate good consistency with the first-principles data published by Liu *et al*.^28^, with observed variations mainly arising from differences in the structural relaxation methods employed.

**Table 2:** Comparison of mechanical properties from this work with literature values.

Material	Property	This Work	Literature
Nb_2_C	E (GPa)	332.41	307^27^
Nb_2_C	G (GPa)	130.06	121^27^
Nb_2_C	υ	0.278	0.273^27^
Mo_2_C	E (GPa)	421.76	390.7^28^
Mo_2_C	G (GPa)	165.96	152.6^28^
Mo_2_C	υ	0.271	0.28^28^

To further validate the accuracy of our structural models, we compared the lattice constant of the pristine MXene structures used in this study with those reported in the literature. As shown in Table 3, our calculated lattice constants are in excellent agreement with previously reported DFT values for the same or closely related crystal structures. This close agreement confirms that our structural relaxations are accurate and that our models reliably represent the ground-state structures of these MXenes.

**Table 3:** Comparison of lattice constant from this work with literature values.

Material	This Work	Literature
Nb_2_C	3.148	3.11^29^
V_2_C	2.883	2.89^30^
Sc_2_C	3.333	3.312^31^
Ta_2_C	3.115	3.106^32^

## Supplementary information

Supplementary Information

## Data Availability

Processed material property data is shared within the Science Data Bank (10.57760/sciencedb.licpdata.00002) in CSV format, where users may browse and download target files independently.

## Code availability

Structural relaxation, electronic properties, mechanical properties, and tribological properties calculations were performed using the VASP code^13^, with TDOS and *E*_*g*_ output via the vaspkit code^24^.
