# Supplementary material for: N-doped MXenes for tribological applications: A high-throughput DFT dataset
Source: Sci Data. 2026 Apr 17;13:893. doi: 10.1038/s41597-026-07236-w (PMC13272584; doi:10.1038/s41597-026-07236-w)
Supplement: Supplementary file 1 — Supplementary Information [file 41597_2026_7236_MOESM1_ESM.pdf]

Supporting Information

N-doped MXenes for tribological applications: A high-throughput DFT dataset

Mingyang Xu, [1, 2] **Yu Gao,** [2, 3, ✉] **Wenhao He,** [2] **Zhongrong Geng,** [1, ✉] **Qian Meng,** [1] **Zhibin Lu**[2, 3]

[1] *School of Materials Science and Engineering, Lanzhou Jiaotong University, Lanzhou 730070, China*

[2] *State Key Laboratory of Solid Lubric ation, Lanzhou Institute of Chemical Physics, Chinese Academy of Sciences, Lanzhou 730000, China*

[3] *Scientific Data Center, Lanzhou Institute of Chemical Physics, Chinese Academy of Sciences, Lanzhou 730000, China*

✉ *Corresponding author: gaoyu@licp.cas.cn (Yu Gao); gzrong-1977@163.com (Zhongrong Geng)*

Table S1. Total energy differences (ΔE = |E_spin-polarized - E_non-spin-polarized|) of the five MXenes with N-doping at site 1.

Material	ΔE (eV)
Nb_2C	6.03×10^{-6}
V_2C	2.06×10^{-6}
Sc_2C	6×10^{-8}
Ta_2C	6.38×10^{-6}
Mo_2C	0.009

Table S2. Bader charge analysis for N-doped V_2C bilayer systems at four doping sites. N_1 and N_2 refer to the N atoms in the upper and lower layers, respectively.

Doping site	Q (N_1) (e)	ΔQ (V_1) (e)	Q (N_2) (e)	ΔQ (V_2)
1	1.293	-5.122	1.347	-5.066
2	1.384	-5.272	1.343	-4.901
3	1.328	-5.180	1.350	-5.077
4	1.294	-5.123	1.347	-5.065

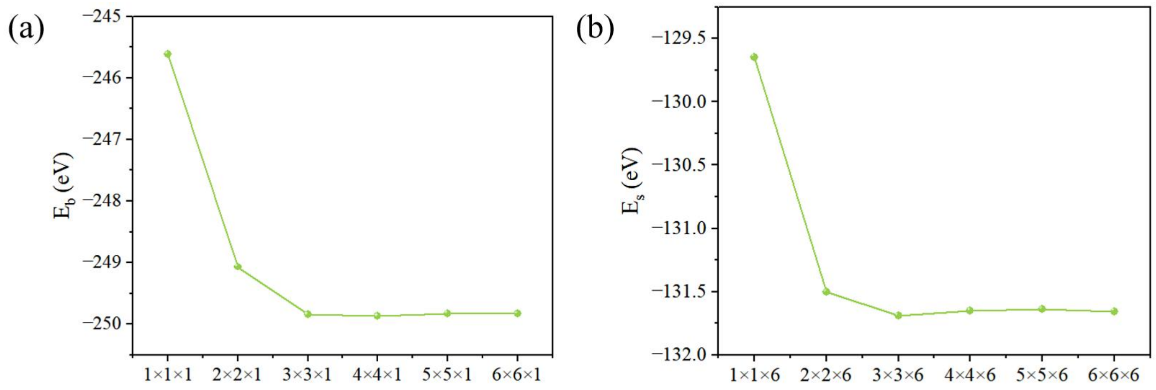

Fig. S1 Convergence tests of total energy with respect to k-point mesh for Nb_2C with doping at site 1. (a) bilayer system tested with k-point meshes from 1×1×1 to 6×6×1, (b) monolayer system tested with k-point meshes from 1×1×6 to 6×6×6.

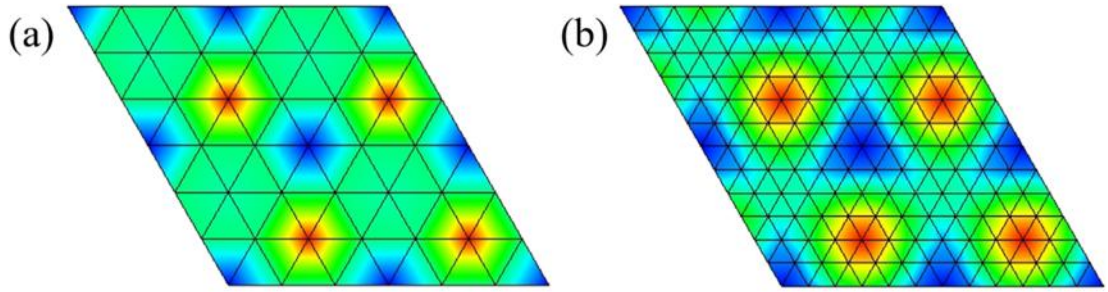

Fig. S2 PES for N-doped Nb_2C at doping site 1 calculated with different in-plane grid resolutions. (a) 6×6 grid, (b) 12×12 grid.

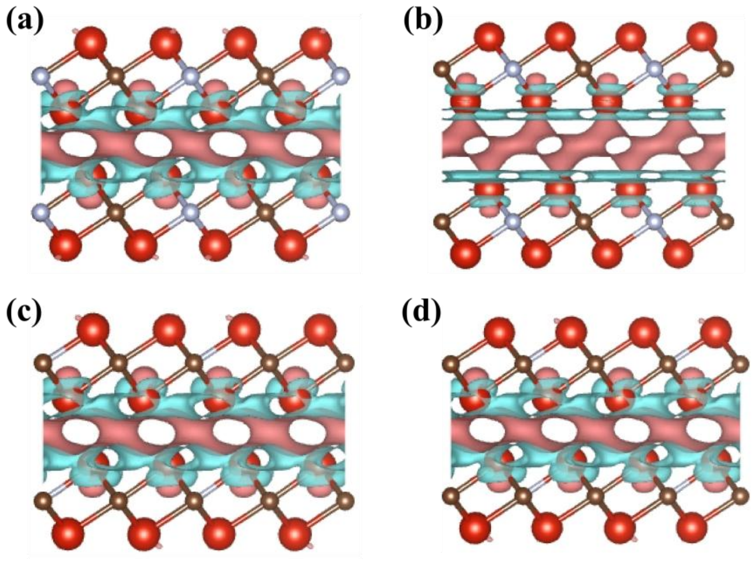

Fig. S3 Differential charge density for N-doped V_2C at different sites. (a) site 1, (b) site 2, (c) site 3, and (d) site 4. Red and cyan isosurfaces represent charge accumulation and depletion, respectively.
